# Rapid Eye Movements in Sleep Furnish a Unique Probe Into Consciousness

**DOI:** 10.3389/fpsyg.2018.02087

**Published:** 2018-10-31

**Authors:** Charles C.-H. Hong, James H. Fallon, Karl J. Friston, James C. Harris

**Affiliations:** ^1^Patuxent Institution, Correctional Mental Health Center — Jessup, Jessup, MD, United States; ^2^Department of Psychiatry and Behavioral Sciences, The Johns Hopkins Hospital, Baltimore, MD, United States; ^3^Department of Anatomy and Neurobiology, University of California, Irvine, Irvine, CA, United States; ^4^Department of Psychiatry and Human Behavior, University of California, Irvine, Irvine, CA, United States; ^5^The Wellcome Trust Centre for Neuroimaging, Institute of Neurology, University College London, London, United Kingdom

**Keywords:** predictive coding, dream, rapid eye movements (REMs) in sleep, autism, visual perception, retrosplenial cortex, claustrum, thalamic reticular nucleus

## Abstract

The neural correlates of rapid eye movements (REMs) in sleep are extraordinarily robust; including REM-locked multisensory-motor integration and accompanying activation in the retrosplenial cortex, the supplementary eye field and areas encompassing cholinergic basal nucleus (Hong et al., 2009). The phenomenology of REMs speaks to the notion that perceptual experience in both sleep and wakefulness is a constructive process – in which we generate predictions of sensory inputs and then test those predictions through actively sampling the sensorium with eye movements. On this view, REMs during sleep may index an internalized active sampling or ‘scanning’ of self-generated visual constructs that are released from the constraints of visual input. If this view is correct, it renders REMs an ideal probe to study consciousness as “an exclusively internal affair” (Metzinger, 2009). In other words, REMs offer a probe of active inference – in the sense of predictive coding – when the brain is isolated from the sensorium in virtue of the natural blockade of sensory afferents during REM sleep. Crucially, REMs are temporally precise events that enable powerful inferences based on time series analyses. As a natural, task-free probe, (REMs) could be used in non-compliant subjects, including infants and animals. In short, REMs constitute a promising probe to study the ontogenetic and phylogenetic development of consciousness and perhaps the psychopathology of schizophrenia and autism, which have been considered in terms of aberrant predictive coding.

## Introduction

Consciousness has long been considered as the brain’s passive response to sensory stimuli from the external world. However, empirical evidence suggests that consciousness is actively generated within the brain: sensory signals from the outside world merely constrain the internal generation of consciousness in wakefulness. Furthermore, in dreaming, the brain generates consciousness without external sensory stimuli. Interestingly, von Helmholtz arrived at the insight in the 19th century: consciousness is an internal and constructive process (von Helmholtz, 1924).

Depicting perception as a process of probabilistic, knowledge-driven inference— as in predictive coding theory — follows Helmholtz’s formulation of perception as inference (1860) (Hobson and Friston, 2012; Clark, 2013; Friston et al., 2014; Hobson et al., 2014). The brain infers (i.e., perceives) the causes of sensations (i.e., the outside world), despite the fact that the brain has no direct access to their source (Clark, 2013). The brain is inferentially secluded from the world and is essentially self-evidencing (Hohwy, 2016). In other words, the brain infers the causes of sensory input hidden beyond the evidentiary boundary afforded by our sensory epithelia (Hohwy, 2016): conscious experience is “an exclusively internal affair” (Metzinger, 2009). For example, nearly 95% of input to the lateral geniculate nucleus is non-retinal (Guillery and Sherman, 2002). According to Llinás and Paré (1991) the brain is essentially a “closed system,” awake or dreaming, which is perturbed and modulated by sensory stimuli. Other theoretical accounts, such as the integrated information theory of consciousness (Tononi et al., 2016), also consider the brain as a closed system and consciousness as an internal affair. This renders REM sleep an ideal state for studying internal brain mechanisms – in isolation from the sensorium – because much of the external sensory input to the brain is blocked in REM sleep (Funk et al., 2016); albeit incompletely (Deiber et al., 1989; Issa and Wang, 2008; Blume et al., 2018). Compared to tonic REM sleep, the sensory blockade is greater during phasic REM sleep, which is characterized by bursts of REMs (Wehrle et al., 2007; Ermis et al., 2010; Windt, 2017). In studies of visual perception in wakefulness, irrelevant non-visual stimuli may confound the interpretation of empirical findings – but this cannot be the case in REM sleep (Hong and Harris, 2009).

Our generative models (explained in section “Hierarchical Predictive Processing — How Does the Brain Infer the World?” as models we use to make sense of our sensorium) pertain not only to the external milieu, but also the bodily-self, as evidenced by recent studies of introception (Blanke, 2012; Ehrsson, 2012) and illucidated by philosophical arguments (Metzinger, 2007). The world-model, with the self-model at its center both in wakefulness (Metzinger, 2007) and dreaming (Staunton, 2001; Hobson et al., 2014; Windt, 2017), can be viewed as an inference architecture, enabling adaptive interaction of the self with the world in simulation (Metzinger, 2009; Windt, 2017). Crucially, multisensory-motor integration time-locked to REMs in sleep (Hong et al., 2009), supports this scientific (Blanke, 2012; Ehrsson, 2012; Hobson et al., 2014) and philosophical (Metzinger, 2007, 2009; Windt, 2017) insight. In short, rapid eye movements (REMs) in sleep may index a fictive (i.e., imaginal or counterfactual) engagement with the world prescribed by a generative model; much like simulations in a virtual world.

**FIGURE 1:**
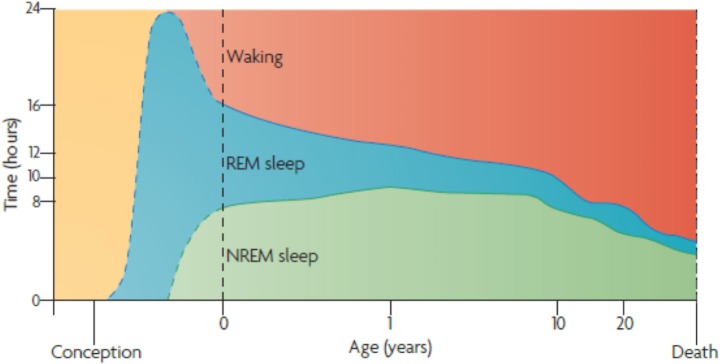
Ontogenesis of REM Sleep. Human sleep and age. The marked preponderance of REM sleep in the last trimester of pregnancy — and the 1st year of life — decreases progressively as waking time increases. Note that non-REM sleep time, like waking time, increases after birth. Despite its early decline, REM sleep continues to occupy approximately 1.5 h per day throughout life. This suggests its strongest developmental contribution is to early brain-mind development; however, it subsequently plays an equally indispensable part in brain-mind maintenance. The ‘after-birth’ part of this figure assimilates findings from several groups (Roffwarg et al., 1966), while the ‘before-birth’ recapitulates Hobson’s extrapolation from a small number of observations of premature newborns (Roffwarg et al., 1966). Adapted from (Hobson, 2009) with the permission from Springer Nature.

There is clear evidence that dreaming and waking perception share the same neural substrates and mechanisms (Hong et al., 1995, 2009; Horikawa et al., 2013; Andrillon et al., 2015). Indeed, Hobson proposed the ‘protoconsciousness theory,’ in which REM sleep has a foundational role in developing and maintaining waking consciousness (Hobson, 2009). Extrapolation from observations of premature newborns (Roffwarg et al., 1966) suggests a marked preponderance of REM sleep in the last trimester of pregnancy (Hobson, 2009) (Figure 1). Indeed, ultrasound imaging – that enables visualization of fetal eye movements – suggest that REM sleep commences *in utero* as early as 23 weeks and constitutes the majority of fetal life into the third trimester of pregnancy (Birnholz, 1981).

Our studies in this area (Hong et al., 2009) have focused on the neural correlates of REMs *per se*, as opposed to phasic REM sleep. Our findings suggest that REM-locked brain activity is distinct from baseline brain activity during phasic REM sleep. Clearly, REM-locked brain activity changes overlap with those associated with REM sleep (particularly, phasic REM sleep, which entails most REMs); however, our findings indicate that the overlap may reflect spillover effects accompanying REMs. In particular, brain activity changes time-locked to *video-timed* REMs speak to the utility of characterizing temporally precise REMs, as opposed to comparing the neural correlates of phasic vs. tonic REM sleep.

Event-related fMRI (characterizing REMs in sleep based on direct visual inspection — as opposed to indirectly by electrooculography) is well placed to capture the neural correlates of consciousness. In this paper, we review how REMs could be considered as indexing the active sampling of a fictive world generated by our internal models (Hong et al., 2009; Hobson et al., 2014). Our fMRI findings provide empirical support for predictive coding, protoconsciousness theory, and related treatments of conscious experience (Hobson et al., 2014). We will review these theoretical constructs and new findings (Andrillon et al., 2015; Auger et al., 2015; Darby et al., 2017) to show how REM-locked sensorimotor integration can reveal some of the fundamental processes that underwrite our experience of the world. In short, we suggest that REMs in dreaming sleep constitute a powerful probe into consciousness — and its normal and abnormal development. To motivate the study of the neural correlates of REMs, we provide a brief introduction to predictive coding theory. We then explain why REMs may offer a unique, promising new probe into consciousness.

## Consciousness As Inference

### How Have Helmholtz’s Insights Been Pursued?

There are two aspects of Helmholtz’s work (von Helmholtz, 1882, 1924), on which neuroscientists have built their theories: perception as inference and the notion of efference copy (von Helmholtz, 1924) (see Box 1). The notion of efference copy was subsequently developed by Sperry (1950), von Holst and Mittelstaedt (1950), Hobson and McCarley (1977), Guillery and Sherman (2002), and Koenderink (2015). In particular, the notions of unconscious inference (von Helmholtz, 1882) and efference copy have been used as the basis of a ‘Bayesian brain’ formulation of sentient or active inference (Friston et al., 2006; Friston, 2009, 2010). In this formulation, the brain infers the causes of its sensations by constructing explanations that minimize surprise (aka prediction error). In this setting, efference copy corresponds to the brain’s predictions of sensory consequences of action. The Bayesian brain is one facet of active inference (Friston, 2009) and the most popular process theory for neuronal implementation of Bayesian inference is predictive coding (Friston et al., 2013). Predictive coding then offers a formal explanation for how the brain makes inferences about the world and, crucially, how false inference can be used to understand psychopathology in conditions like autism (Pellicano and Burr, 2012; Friston et al., 2013, 2014; Gómez et al., 2014; Lawson et al., 2014; Quattrocki and Friston, 2014; Skewes et al., 2014; Van de Cruys et al., 2014) and schizophrenia (Dima et al., 2009; Fletcher and Frith, 2009; Brown et al., 2013; Friston et al., 2014).

**Box 1. Corollary discharge**

A copy of motor commands is sent to sensory processing regions of the brain. Corollary discharge is crucial for the control of subsequent movements (particularly for rapid sequential movements, like saccades in waking or REMs in sleep) and for the interpretation of subsequent sensory information. Without the suppression of visual motion cues during movements (a.k.a. saccadic suppression) through corollary discharge, REMs would confound vision. This is an important example of sensory attenuation.

**Efference copy**

An internal copy of an efferent motor command: von Helmholtz, a German physician and physicist, first proposed the notion of efference copy to explain why passive movement of the eyeball – on pressing it – causes a percept that the world moves. In contrast, volitional movements of the eyeball – caused by motor commands – ensure that we perceive the world as remaining still. Efference copies may therefore play a key role in saccadic suppression, or sensory attenuation, and may also explain why we cannot tickle ourselves. Under predictive coding formulations, efference copy is equivalent to corollary discharge, mediating top-down predictions and sensory attenuation.

Hobson and McCarley pursued Helmholtz’s suggestion that efference copies of motor commands are sent to the sensory processing areas to predict the sensory effects of movement, in both waking and dreaming (von Helmholtz, 1924). Indeed, firings of burst neurons in the brainstem consistently precede ipsiversive REMs and ponto-geniculo-occipital (PGO) waves. This suggests that PGO waves convey the direction of eye movements (efference copy or corollary discharge) to visual sensory systems (Nelson et al., 1983). In short, Hobson and McCarley (1977) proposed that the cyclically activated forebrain synthesizes dreams from internally generated information.

### Do We Perceive the World as It Is?

According to Metzinger nature’s virtual reality world-model can be viewed as “online simulation” (Metzinger, 2009). “Millions of years ago, nature’s [own neural] virtual reality [machine] achieved what today’s software engineers still strive for: the phenomenal properties of presence and full immersion” (Metzinger, 2009). These properties convince us that what we perceive in wakefulness is the world itself (dubbed as ‘naïve realism’). But what we perceive is a model of the world generated by the brain. The brain is both a hierarchical and parallelized system that is able to infer the world; it can source the causes of limited data that are sampled by our sensory organs (Friston, 2010; Clark, 2013). The experience of color is a compelling example, demonstrating that the real world ‘out there’ can be differentiated from what we perceive – and also demonstrating the limits of our perceptual capacity. Our brain can process only a tiny portion of the electromagnetic spectrum ‘out there’ that includes gamma ray, X-ray, ultraviolet, ‘visual spectrum,’ infrared, microwave, and radio wave frequencies. The color we perceive is not “out there” in the real world. “It is a property of the internal model … created by your brain” (Metzinger, 2009). The only things out there are the reflectance properties of objects and electromagnetic waves of varying wavelengths. The real world is colorless, but the brain paints the internal model with colors to encode differences in wavelengths of the ‘visual spectrum’ (400–700 nm) (Metzinger, 2009).

One caveat is needed in using the popular term ‘virtual reality.’ We use this expression to underline the fact that what we consciously experience to be the world is not the world itself, but the content of a model of the world generated *from within*; we do not mean that the world we live in is virtual, not real, or that there is no underlying physical reality behind our perception (Metzinger, 2009).

### A Virtual Body at the Center of a Virtual Reality in Wakefulness

**FIGURE 2:**
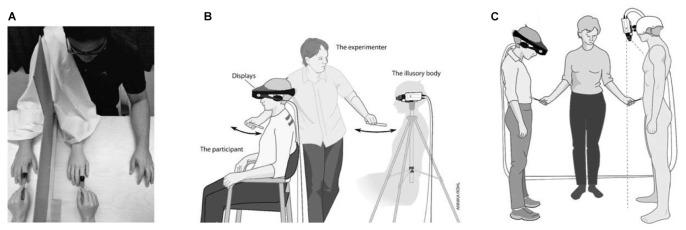
Rubber hand illusion, out-of-body illusion and mannequin illusion. **(A)** Experimental setups used in the rubber hand illusion. For details, see the text. In **(B,C)** the participants wear a set of head-mounted displays connected to two video cameras placed on a tripod 1.5 m behind the participants in **(B)** or on the mannequin’s head in **(C)**. The images from the left video camera are presented on the left eye display and the images from the right camera on the right display. In **(B)** the participants see themselves from the point of view of the cameras, that is, from behind. In **(C)** the cameras are positioned so that the participants are looking down on the mannequin’s body with stereoscopic vision. Adapted from Ehrsson (2012) with permission from the MIT Press.

If the world we perceive is a virtual reality, what about the body that we perceive to be our own? Interestingly, the rubber-hand illusion (Botvinick and Cohen, 1998) and experimentally induced out-of-body experiences (Ehrsson, 2007) indicate that what we perceive as the bodily self in wakefulness is also a construct of the brain (Metzinger, 2007; Blanke, 2012; Ehrsson, 2012) (Figures 2A,B). Furthermore, these experiments suggest that multimodal sensory integration is essential for generating a model of the bodily self (Metzinger, 2007; Blanke, 2012; Ehrsson, 2012). Viewing a rubber hand being stroked with a paintbrush, while feeling (synchronous but unseen) touch sensations from the corresponding part of the real hand generates the illusion that the touch sensations emanate from the rubber hand (Botvinick and Cohen, 1998) – and even induces experience of illusory ownership of the fake hand (Metzinger, 2007; Blanke, 2012; Ehrsson, 2012). Indeed, seeing the fake hand being injured causes people to flinch with attendant and marked physiological responses (Metzinger, 2007; Ehrsson, 2012). A whole body version of the classic rubber hand illusion employs a mannequin and a head-mounted display to provide synchronous visual and tactile stimuli (Metzinger, 2007; Petkova et al., 2011; Ehrsson, 2012) (Figure 2C).

Crucially, at least 20% of persons born without limbs (phocomelia) experience phantom limbs, indicating that the bodily self-model can be innately generated by neural networks, without sensory input from the limbs (Melzack et al., 1997; Metzinger, 2003). The bodily self-model — being in part genetically determined — likely serves this fundamental function (Melzack et al., 1997; Metzinger, 2003). We will return to this later. The world-model, with the self-model at its center, can be viewed as a highly “advanced, user-friendly interface design,” and “a wonderfully efficient control device,” “like a total flight simulator” (but whose “virtuality is hidden”) in which our bodily self manipulates the virtual environment in wakefulness (Metzinger, 2009), and, of course, in dreaming.

### REM-Locked Generation of a Virtual Body

The key contribution of REM-based fMRI studies is evidence for multisensory integration time-locked to REMs in dreaming sleep (Hong et al., 2009). Multimodal sensory integration is essential for generating a bodily self-model (Metzinger, 2007; Blanke, 2012; Ehrsson, 2012). Thus, REMs may be associated with the generation of (bodily somatosensory) self-images in dreaming (Hobson et al., 2014). Interestingly, the brain regions implicated in construction of a bodily self-model in wakefulness (Blanke, 2012) are also found to be activated in a time-locked manner to REMs in sleep; namely, primary somatosensory cortex, premotor cortex, vestibular cortex, insula, anterior cingulate cortex, putamen, and superior temporal gyrus (Hong et al., 2009). REM-locked *multisensory integration* seems to be an integral part of generating both a virtual reality and a virtual body in dreaming.

The similarities between waking and dreaming consciousness go beyond perception of the surrounding world or bodily self, and extend to *interactions* between them. Dreams are almost always from the first-person perspective (Staunton, 2001; Hobson et al., 2014) and the self is in the perceived center in dreaming, not only as an observer (Staunton, 2001), but also as an agent, a virtual body that navigates a virtual reality in dreaming (Hobson et al., 2014). The virtual body is embodied at the center of virtual reality, as an agent, both in wakefulness (Metzinger, 2007) and in dreaming. Hobson (2009, 2017) proposed that this body-world interaction is simulated in REM sleep, which is copious *in utero*, to promote the survival of our physical body in the physical world after birth.

### Hierarchical Predictive Processing — How Does the Brain Infer the World?

Perception entails inference or modeling (Hobson et al., 2014). Modern-day versions of Helmholtz’s formulation of perception consider the brain as a ‘Bayesian inference’ machine (Friston, 2010; Clark, 2013). Bayesian inference is a method of statistical inference, in which the prior probability for a hypothesis (often simply called a ‘prior’) is updated as data become available. The brain has prior beliefs (hypotheses or predictions) about states of the world ‘out there,’ before sampling their sensory consequences. For example, the brain has prior knowledge that faces are convex and — in the hollow mask illusion — this prior is typically strong enough to override veridical sensory signals generated by a concave facial mask. Predictive coding theory explains how the brain perceives the world, i.e., how the brain infers the world hidden in sensory data (Rao and Ballard, 1999; Friston, 2010; Friston et al., 2012, 2014; Clark, 2013). This theory provides a computational or formal explanation for how the brain generates its model of the world; i.e., how we “infer the nature of the signal source (the world) from just the varying input signal itself” (Clark, 2013). A ‘*generative model*’ is a probabilistic model used in statistics and in machine learning to estimate the hidden causes underlying observed phenomena. It is usually specified in terms of a prior belief about the causes and the likelihood of observations, given the causes (Friston, 2010; Hobson and Friston, 2014). On this view, perceptual synthesis corresponds to selecting hypotheses that explain sensations (Gregory, 1980). In brief, the brain generates a hypothesis about the causes of sensory data (i.e., ‘prediction’ in predictive coding) and then tests the hypothesis by comparing it with sampled sensory data to check for any discrepancy (i.e., ‘prediction error’ in predictive coding).

**FIGURE 3:**
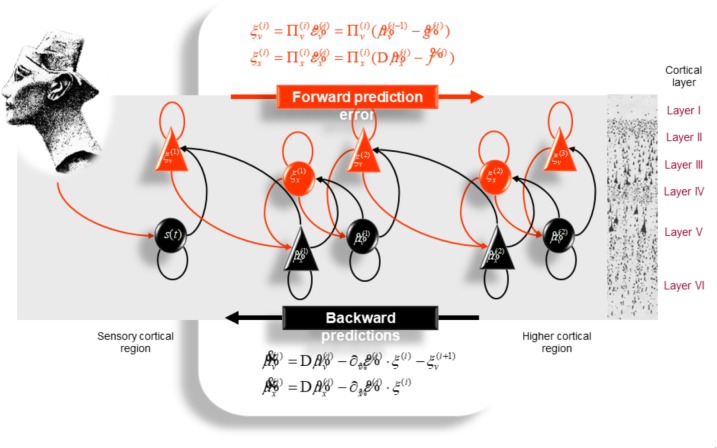
Schematic representation of the neuronal architecture that might encode conditional expectations about the states of a hierarchical model. This shows the speculative cells of origin of forward driving connections that convey prediction error from a lower area to a higher area and the backward connections that construct predictions. These predictions try to explain away prediction error in lower levels. In this scheme, the sources of forward and backward connections are superficial and deep pyramidal cells, respectively. The equations represent a gradient descent on free-energy under the hierarchical models described in Friston et al. (2012). State units are in black and error units in red. Here, neuronal populations are deployed hierarchically within three cortical areas (or macro-columns). Within each area, the cells are shown in relation to cortical layers: supra-granular (I–III) granular (IV) and infra-granular (V–VI) layers. For simplicity, conditional expectations about control states had been absorbed into conditional expectations about hidden causes. Adapted from Friston et al. (2012) with permission from Friston.

It is generally thought that the brain embodies a *hierarchical* generative model (Friston et al., 2012; Hobson and Friston, 2012; Hobson and Friston, 2014; Hobson et al., 2014). In predictive coding (Rao and Ballard, 1999; Friston, 2010; Clark, 2013), the brain infers the cause of sensory signals (ultimately, the world) by updating its internal model of the world through a multilevel cascade of processing that involves recurrent message passing between higher and lower levels of the cortical hierarchy (Figure 3). “In hierarchical models, the output of one level acts as an input to the next” (Friston et al., 2012). Our brains have a *hierarchically deep* structure, which enables the brain to acquire models of increasing hierarchical depth, progressively higher or deeper levels of representational abstraction or explanation (Hobson et al., 2014). The higher in the hierarchy, the more integrated and abstract – and the further removed from sensory data the empirical priors become. This is in line with the following description of “mathematical elegance” reflected in the structure of the natural world (Davies, 1992): “The essence of Life’s complexity lies not with the rules, but with their repeated use. The computer has to work hard applying the rule again and again, before it can generate deeply complex patterns from simple initial rules… The study of computation has enabled us to recognize that the world is ordered both in the sense of being algorithmically compressible, and in the sense of having depth.”

Efficient statistical inference is evident in the retina, at the initial stages of sensory information processing by the brain (Hosoya et al., 2005). Predictive coding equips the brain with an efficient perceptual tool to generate a model of the world hidden behind sensory data. Clearly, our brain has to explain the world outside quickly and efficiently; otherwise, we could not survive in a capricious and changeable world. Predictive coding theory explains how the brain accomplishes this — and what might happen if inference goes awry. We will return to this in Section “Active Inference and Protoconsciousness Hypothesis.”

### Predictive Coding Theory May Explain Self-Modeling

If predictive coding theory explains how the brain infers and generates models of the world, why not models of the bodily self? (Limanowski and Blankenburg, 2013). In brief, active inference and predictive coding have the potential to explain how the brain infers and generates its model of bodily self from visual, tactile, proprioceptive and vestibular sensory data; that is, “by predictive multisensory integration of self-related signals (Seth, 2013).” On this view, one might expect that predictive coding can also explain what happens when this predictive integration fails. That is, the distorted, often delusional, perception of bodily self in anorexia nervosa and body dysmorphic disorder may be caused by defective bodily-self-modeling, due to aberrant predictive coding. A review of the literature on visual processing in anorexia nervosa and body dysmorphic disorder suggests over-attention to detail and reduced processing of global features – both in anorexia nervosa and body dysmorphic disorder (Madsen et al., 2013). Studies of non-visual (tactile, proprioceptive and interoceptive) sensory processing in anorexia nervosa also show a similar impairment (Gaudio et al., 2014). For example, individuals with anorexia nervosa overestimate the size of tactile distances, and this false inference correlates with psychopathology (Keizer et al., 2011). Anorexia nervosa patients experience the rubber hand illusion more acutely than controls, which also correlates with psychopathology (Eshkevari et al., 2012). Interestingly, according to the predictive coding theory, aberrant inference about the (exteroceptive) world and the (interoceptive) bodily self offers a plausible explanation for autistic or schizophrenic experience (as expounded later in section “Predictive Coding Accounts of Severe Mental Disorders”).

### Aberrant Self-Modeling

Patients with Cotard delusion deny existence of the self (and frequently even the world) (Berrios and Luque, 1995a; Metzinger, 2003/2004). The most common theme of Cotard delusion involves bodily self (Berrios and Luque, 1995b). The Cotard delusion suggests that what we consciously experience to be ourselves is also the content of a model generated by our brain (Metzinger, 2003/2004). Self-modeling may go awry, just as aberrant world-modeling may be manifest in schizophrenia. Delusional misidentification of other persons (Capgras delusion) and places (reduplicative paramnesia) is related to delusions about self (Cotard delusion) (Förstl et al., 1991). Capgras delusion is the belief that a close relative or spouse has been replaced by an identical-looking impostor. Reduplicative paramnesia involves the delusional belief that a place or location has been duplicated. Delusional misidentification (delusion of doubles) of pets and inanimate objects was also reported (Berson, 1983; Anderson and Williams, 1994; Wright et al., 1994; Ellis et al., 1996; Darby and Caplan, 2016). Prosopagnosia consists in the inability to recognize *familiar faces* (Duchaine and Nakayama, 2006; Corrow et al., 2016), “including, in some cases, one’s *own* face reflected in a mirror” — personal identity as visually given disappears from conscious experience (Metzinger, 2003).

These delusions and prosopagnosia are rare but coexist in some cases, and are commonly associated with brain diseases (Berson, 1983; Anderson, 1988; Förstl et al., 1991; Anderson and Williams, 1994; Weinstein, 1994; Wright et al., 1994; Ellis and Lewis, 2001; Debruyne et al., 2009). This indicates a shared neural infrastructure that constitutes the generative model of self and world – including other persons, pets, objects and places (and underlies familiarity perception) (Darby et al., 2017). Indeed, a recent study (Darby et al., 2017) identified such an overlap in the left RSC; namely, they showed that the left RSC was the region most likely to be activated by *personally familiar* (versus unfamiliar) sensory (visual and auditory) stimuli (persons, places, and objects) (Darby et al., 2017).

### Retrosplenial Cortex

The RSC contributes to autobiographical, episodic memory as well as spatial memory and navigation (Maguire, 2001; Harker and Whishaw, 2004; Vann et al., 2009). Autobiographical memo comprises episodes of self in a place (i.e., backdrop) and thus involves spatial processing. The RSC codes specifically for permanence of location among different attributes of various outdoor items (Auger et al., 2015), and this coding for permanence may be a prerequisite for successful navigation (Auger et al., 2012; Auger and Maguire, 2013) and for scene processing and autobiographical memory (Auger et al., 2015). Problems with permanence coding by RSC may explain spatial disorientation and episodic memory impairment — the early signs of Alzheimer’s disease (Auger et al., 2012, 2015; Auger and Maguire, 2013). Interestingly, RSC hypometabolism (Minoshima et al., 1997; Nestor et al., 2003) and atrophy (Pengas et al., 2010) have been found at its earliest stages (Vann et al., 2009 for review).

#### Neural Correlates of Self-Modeling, Familiarity Perception and Spatial Navigation

Both delusional misidentification syndrome and familiarity perception were found to be linked to the left RSC (Darby et al., 2017) and there are apparent connections between the two. Capgras delusion targets “those people to whom the patient was tied with intense affective sentiment” (Capgras et al., 1924), mostly parents and spouses (Berson, 1983). All delusional misidentification concerns personally familiar objects. It has been suggested that both Capgras delusion and prosopagnosia result from disturbance of integrating or imbuing visual perception with affective familiarity (Anderson, 1988). A person with Cotard delusion finds it “impossible to consciously experience *familiarity* with himself” (lose “self-intimacy”); “the physical body is not *owned* anymore” (Metzinger, 2003/2004). Familiarity or intimacy with self is crucial for adaptive interaction of the self with the world and self-preservation in the world (Metzinger, 2003/2004).

In summary, RSC activity is correlated both with permanence coding (positively) and with familiarity perception (and aberrant bodily-self-modeling) (negatively, with left RSC). The bodily self-model is “the *spatial* model of the self” (Metzinger, 2003). We propose that RSC plays a crucial role in the aforementioned innate generation of the spatial bodily self-model (Melzack et al., 1997; Metzinger, 2003). This proposal is supported by RSC’s role in spatial permanence coding (Auger et al., 2012, 2015; Auger and Maguire, 2013), the permanently central location of the bodily self-model (except in out-of-body experience), RSC’s involvement in Cotard’s syndrome (Darby et al., 2017), and the early neurodevelopmental establishment of RSC in neural circuits. For example, the default mode network (DMN) can be seen in children of ages 2 weeks to 2 years; where the RSC is the main hub of the emerging DMN (Gao et al., 2009). Neurobiological findings further suggest that the RSC is a key structure in normal and abnormal self-modeling, familiarity perception and spatial navigation (permanence coding). In short, the RSC appears to play a central role in self-modeling (Metzinger, 2003/2004) and predictive coding formulations of self-world interactions (Hobson and Friston, 2012, Friston, 2014; Hobson et al., 2014).

#### REM-Locked Peak Activity in the Right Retrosplenial Cortex

**FIGURE 4:**
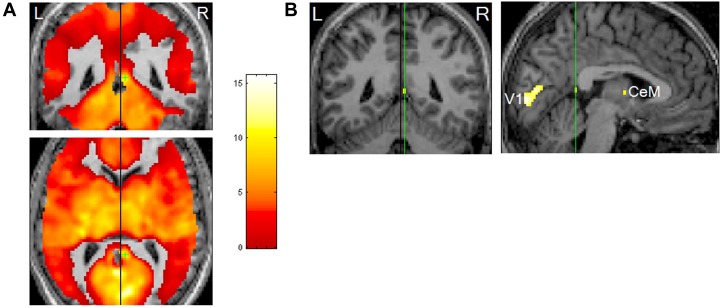
Rapid eye movements (REM)-locked activation in retrosplenial cortex (RSC). **(A)** Upper panel shows coronal view, lower axial view. Black lines indicate the mid-sagittal plane. Green crosshair is at the right RSC peak activity point (Talairach coordinates 4, –46, 12, *t* = 10.5). Threshold has been reduced to uncorrected *P* < 0.5 (*t* = 0) to illustrate the failure to detect significant responses in the RSC on the left hemisphere, which contrasts with the adjacent robust activation in the right hemisphere. **(B)** Left panel shows a coronal view and right sagittal view. The black line on the coronal view indicates the mid-sagittal plane. Green lines pass the right RSC peak activity point and show location of the other views. Thresholded higher at corrected *P* < 0.00005 (*t* = 10.1). V1, primary visual cortex; CeM, central medial thalamic nucleus (a part of intralaminar/midline group). Adapted from Hong et al. (2009) with the permission from Hong.

Notably, the RSC is an area that shows pronounced REM-locked activity changes —robust REM-locked activation has been observed in the right RSC, in contrast to an adjacent area (with no statistically significant activation) in the left RSC (Hong et al., 2009) (Figure 4). This finding is remarkable because RSC is relatively small and the left and the right RSCs border each other. This finding attests to the capacity of fMRI of video-timed REMs to localize REM-locked activation (Hong et al., 2009). Our fMRI study of video-timed REMs (Hong et al., 2009) and Darby et al. (2017), along with lesion studies that suggest that the right RSC is involved in topographic orientation or spatial navigation (Maguire, 2001), speak to the importance of comparing the left RSC and the right RSC in future studies (see Figure 4).

## Neural and Perceptual Correlates of REMS During Dreaming Sleep

**FIGURE 5:**
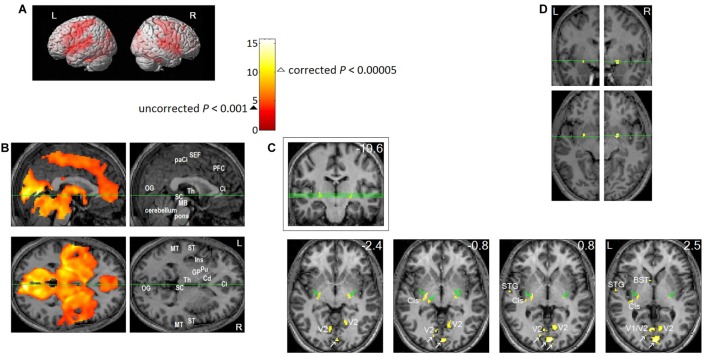
Brain regions activated in association with REMs in sleep. Eleven healthy participants participated in the study. Each participant slept two consecutive nights in the MRI scanner from about 11 pm until they awoke in the morning. We obtained fMRI data as soon as REMs were recognized by video-monitoring, and for as long as they continued. Twenty four REM sleep scans were obtained from eleven participants. We timed REMs from video recording, then to study the neural correlates of REMs, we employed a rapid event-related Blood Oxygenation Level Dependent (BOLD) fMRI design, where each REM constituted a natural event. From the timing of REMs, we predicted the time course of fMRI signal (estimated hemodynamic changes following REMs), then identified the voxels that match this predicted time course using a general linear model. Then, we performed ‘random effects’ analysis on the results of the 24 individual scans (one sample *t*-test). We used SPM2 for these statistical analyses. All images were spatially normalized. **(A,B)** Thresholded at uncorrected *P* < 0.001 (*T* = 3.5, 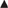); an additional spatial extent threshold of five continguous voxels was applied. **(A)** Projection onto a surface rendering of a template brain. This shows activation of precentral motor cortex and postcentral somatosensory cortex time-locked to REMs. **(B)** Upper row shows median sagittal views, lower row axial views. Green lines show location of the other views. This shows that REM-locked activation is characteristically widespread. Ci, anterior cingulate gyrus; Cd, head of caudate nucleus; GP, globus pallidus; Ins, insula; MB, midbrain; MT, middle temporal gyrus; OG, occipital gyrus; paCi, paracingulate gyrus; PFC, medial prefrontal cortex; Pu, putamen; SC, superior colliculus; ST, superior temporal gyrus; Th, thalamus. See Figure 3 in (Hong et al., 2009) for REM-locked activation (corrected *P* < 0.05) of non-visual primary sensory cortices (auditory, vestibular, and olfactory), Wernicke’s and Broca’s area, fusiform gyrus and mesopontine tegmentum. **(C,D)** To highlight peak REM-locked activations, the threshold was raised to a high level, corrected *P* < 0.00005 (*T* = 10.1, Δ). **(C)** Peaks were clearly localized in striate cortex (the primary visual cortex, V1. White arrows indicate V1), V2, thalamic reticular nucleus (indicated by green arrows), claustrum (Cls, the posterior-ventral zone, i.e., the putative visual zone), superior temporal gyrus (STG), bed nucleus of stria terminalis (BST). Green lines on the coronal view inside the box (the number in mm denote distance from the AC) show locations of the serial axial views (the numbers in mm denote distance from the AC-PC plane). **(D)** Peaks were clearly localized to the substantia innominata, bilaterally. These clusters overlap the cholinergic basal nucleus projecting to the posterior neocortex, including visual cortex. Adapted from Hong et al. (2009) with the permission from Hong.

It should be noted that functionally segregated responses were based on the correlates of rapid *eye movements* (i.e., changes in brain activation when eye movements occur) during REM sleep (Hong et al., 2009), not the correlates of the REM sleep episode, which includes substantial portions without eye movements [73–86% of REM sleep is devoid of REMs (Arnulf, 2011)]. The time resolution of fMRI (seconds) is suitable for studying the neural correlates of eye movements where brain activation associated with REMs in sleep is *characteristically widespread* (Hong et al., 2009) (see Figure 5B).

### REM-Locked Binding

Surprisingly, the peaks of the REM-locked activation were clearly localized not only in the primary visual but also in the primary non-visual sensory cortex — and regions implicated in perceptual binding of elemental attributes of visual perception (shape, movement, and color) and of visual and non-visual perception to form a unified percept (Figure 5). The neuronal correlates of binding have been studied extensively in wakefulness (Steriade et al., 1991; Tallon-Baudry and Bertrand, 1999; Engel and Singer, 2001; Logothetis et al., 2001; Niessing et al., 2005) — and in REM sleep (Llinás and Ribary, 1993), and particularly when *eye movements* occur in REM sleep (Gross and Gotman, 1999; Jouny et al., 2000). These studies and ours (Hong et al., 2009) support the notion that synchronous gamma oscillations mediate binding both in wakefulness and dreaming. However, it cannot be ruled out that such REM-locked gamma oscillations may have contributions from eye movements (Hipp and Siegel, 2013; Muthukumaraswamy, 2013; Yuval-Greenberg et al., 2008).

### REM-Locked Peak Activation in the Thalamic Reticular Nucleus and the Claustrum

Based on neuroanatomy and the macroscopic organization of neural structures, Crick and Koch proposed the thalamic reticular nucleus and claustrum as two candidates crucial for binding information distributed within and across different sensory and motor modalities in the brain (Crick, 1984; Crick and Koch, 2005). It was remarkable that robust peak activation time-locked to REMs were identified within the thalamic reticular nucleus and claustrum (Figure 5C) (Hong et al., 2009). In 2017, Koch reported a new digital reconstruction method that enabled the tracing of three neurons in the claustrum of a mouse - and these neurons arborize extensively throughout the cortex across both hemispheres (Reardon, 2017). Interestingly, a recent DTI study - of a hundred human subjects - showed that that the claustrum is the most densely connected area among all the brain regions studied (Torgerson et al., 2014). The role of the claustrum in binding and salience detection has also been considered by Remedios et al. (2010) and Smythies et al. (2012), respectively. Electric stimulation through an electrode implanted in the claustrum in a female caused reversible disruption of consciousness; complete arrest of volitional behavior, unresponsiveness and no recollection of the event (Koubeissi et al., 2014). In summary, convergent evidence suggests that the claustrum plays a crucial role in sensorimotor integration and generation of consciousness in waking (Crick and Koch, 2005; Remedios et al., 2010; Smythies et al., 2012; Koubeissi et al., 2014; Torgerson et al., 2014; Reardon, 2017) and dreaming (Hong et al., 2009).

### Correlates of Waking Visual Perception and Imagery and Dream Visual Imagery

#### Shared Neural Machinery for All Visual Experience

**FIGURE 6:**
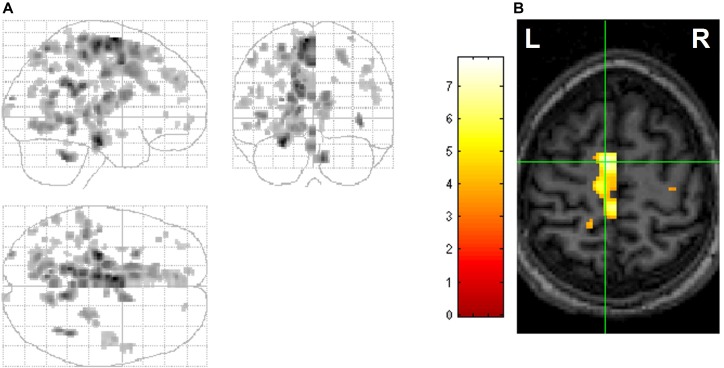
Hemispheric differences in REM-locked activation. Voxels with greater REM-locked activity compared to its homolog in the other hemisphere are shown. All images are thresholded at uncorrected *P* < 0.001 (*T* = 3.5) and additionally at a spatial extent of >5 contiguous voxels. **(A)** Orthogonally oriented statistical parametric maps (maximum intensity projections). REM-locked activation was greater in the posterior left hemisphere. **(B)** Cross at the greatest hemispheric difference, supplementary eye field (SEF) (left greater than right). The left supplementary eye field has a clearly predominant role in sequencing saccade programming during visual scanning in wakefulness. Adapted from Hong et al. (2009) with the permission from Hong.

Importantly, persons with damage to the posterior left hemisphere not only lose the capacity to dream, but also lose capacity to generate waking visual imagery from long-term visual memory (Farah, 1984). A study of split-brain patients (whose corpus callosum connecting the two hemispheres of the brain is surgically severed to treat refractory epilepsy) also endorses the left posterior localization for the image generation process (Farah et al., 1985). Greater REM-locked activation in this region (Hong et al., 2009) (Figure 6A) gives additional support that machinery is shared for generation of waking and dreaming visual imagery. Interestingly, the review of PET and fMRI studies concluded that waking visual perception also shares the same neural machinery with waking visual imagery (Kosslyn et al., 2001). In summary, the machinery is shared regardless of presence (waking visual perception) or absence (waking and dream visual imagery) of visual signals from outside world.

#### Scanning Eye Movements Play a Generative Role for All Visual Experience

In addition to findings suggesting that machinery is shared for all visual experience, there is evidence for the shared role of scanning eye movements in the generation of all visual experience. Studies with split-brain patients suggest that objects in images are constructed sequentially, one part at a time, and parts are arranged into a proper configuration in the left cerebral hemisphere (Kosslyn, 1988). These findings – revealing the sequential construction of parts to form waking visual imagery – are consistent with the findings of scanning eye movements in the generation of waking visual imagery (Brandt and Stark, 1997; Laeng and Teodorescu, 2002). The scanpaths (repetitive sequences of fixations and saccades) during waking visual perception (see Box 2) are highly correlated with those during waking visual imagery of the same visual object; suggesting that scanpaths are an integral part of the visual representation – and are used as a spatial index for the construction of component parts of an image during image generation (Brandt and Stark, 1997; Laeng and Teodorescu, 2002; Hassabis and Maguire, 2009; Mirza et al., 2016). Hebb (1968) hypothesized that “such an empty gaze, serves the function of assisting the mental re-construction of a representation” (Laeng et al., 2014). Here, empty gaze implies saccadic fixation that cannot garner any visual information. Likewise, in dreaming, REMs may reenact the scanpath of eye movements in waking visual perception of the same object or scene and retrieve the visual information encoded by the scanpath (Hong et al., 2009).

**Box 2. Saccadic eye movements**

To enable waking visual perception, the eyes move quickly from one fixation point to the next. These scanning, jerky eye movements are called saccades. The brain automatically chooses points to scan that have salient or precise information, e.g., another’s eyes and lips, and sample visual sensory data from those points. Saccades are necessary for data sampling, because the part of the retina which provides high-resolution visual sensory data (called fovea) is very small in human and sees only the central two degrees of the visual field. The distributed neuronal circuit that controls saccadic eye movements and subserves visuospatial attention (called the oculomotor circuit) has been extensively studied in humans and animals (see Leigh and Zee, 2015 for review). There has been some controversy over whether REMs are saccades. We demonstrated, with PET (Hong et al., 1995), and functional MRI (fMRI) (Hong et al., 2009), that REMs are also saccades, which are associated with activation of the oculomotor circuit, in a time-locked manner (using fMRI that has superior time resolution). Scanning saccadic eye movements in wakefulness occur about four times a second, which interestingly is about the same frequency of bursts of PGO waves and REMs in sleep. This figure shows a scanpath during free examination of a photograph with both eyes for 1 min. Adapted from (Yarbus, 1967) with the permission from Springer United States.

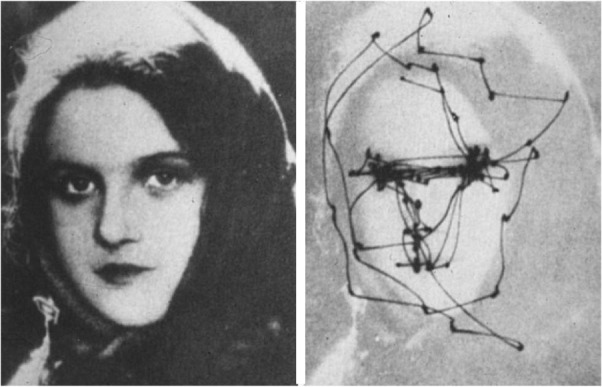

A synthesis of the findings of others (Farah, 1984; Kosslyn, 1988; Brandt and Stark, 1997; Kosslyn et al., 2001; Laeng and Teodorescu, 2002; Friston et al., 2012) and our own work (Hong et al., 2009) suggests that neuronal machinery is shared for waking visual perception, waking visual imagery and dream visual imagery. It further suggests that scanning eye movements play a role in the generation of images in all of these contexts. This perspective of the generic image-generating role of scanning eye movement fits nicely with the ‘active inference’ (Brown et al., 2013) and predictive coding theory (Figure 3). From this perspective, our findings of REM-locked activation in the supplementary eye field (SEF) in the left hemisphere (Hong et al., 2009) (Figure 6B) can be taken not only as evidence that REMs scan dream visual imagery but also as evidence that REMs generate dream visual imagery. The left SEF has a predominant role in sequencing saccadic eye movements in waking visual perception (Gaymard et al., 1993; Grosbras et al., 1999, 2001).

### Single Unit Study of the Neural Correlates of REMs

Andrillon et al. (2015) measured single-neuron activity and intracranial electroencephalography (EEG) time-locked to REMs in neurosurgical epilepsy patients. This study demonstrated that REMs in sleep are associated with *hierarchical* processing of *visual* information (Andrillon et al., 2015). Electrode locations were chosen exclusively for clinical purposes but included key regions in visual processing; namely, hippocampus, entorhinal cortex and parahippocampal gyrus. These are MTL structures belonging to the ventral visual pathway, also known as the ‘what pathway’ and are known to be associated with visual-mnemonic processing. Researchers measured changes time-locked to REMs in sleep and, for comparison, visual and non-visual REMs in wakefulness and visual presentation of famous people and landmarks during fixation without eye movements. They found that “Individual neurons, especially in the MTL, exhibit reduced firing rates before REMs as well as transient increases in firing rate immediately after, similar to activity patterns observed upon image presentation during fixation without eye movements. Moreover, the selectivity of individual units is correlated with their response latency, such that units activated after a small number of images or REMs exhibit delayed increases in firing rates.”

Single unit (neuron) recording or deep event-related potential can directly measure 10 ms difference in latency unlike fMRI, but has limited regional coverage. fMRI has limited time-resolution (seconds), but it can perform simultaneous regional measurement in the whole brain. Thus, our fMRI study of video-timed REMs could guide selection of electrode locations for future single-neuron recording studies of the neural correlates of REMs. Thalamic reticular nucleus, claustrum, cholinergic basal nucleus, and primary visual cortex (Figure 5C) and RSC (Figure 4B) are promising locations to study. The strategy of identifying study location with fMRI before single unit recording worked well to elucidate face recognition (Chang and Tsao, 2017).

### REM-Locked Visual and Hierarchical Processing

Considering findings that used complementary methods (Hong et al., 2009; Andrillon et al., 2015), we conclude that information processing time-locked to REMs is *visual* and *hierarchical* (Hobson et al., 2014). Our fMRI findings clearly showed REM-locked processing of *visual* information. We found the most robust REM-locked activation localized to the primary visual cortex, the ‘visual thalamic reticular nucleus’ and the ‘visual claustrum’ (Figure 5C) (Hong et al., 2009). REM-locked multisensory integration (Hong et al., 2009) suggests that REM-locked processing is *hierarchical* (Hobson et al., 2014). Obviously, prior to multisensory integration, each sensory modality should be processed first in its respective primary sensory cortex. In other words, the output of the primary sensory cortex acts as an input to higher hierarchical level that binds multisensory information. REM-locked activation of Broca’s and Wernicke’s language areas (Hong et al., 2009), which belongs to a higher hierarchical level than the primary sensory cortex, also suggests that REM-locked information processing is hierarchical (Hobson et al., 2014). Our findings (Hong et al., 2009) speak to REM-locked hierarchical processing, which is an essential component of the predictive coding hypothesis (although direct evidence for REM-locked prediction or prediction error signals, another essential component of predictive coding, is still lacking).

### Controversy About the Scanning Hypothesis

A broad body of data supports the idea that REMs scan dream imagery, but controversy about the implicit scanning hypothesis remains. See (Arnulf, 2011) for review. Several authors have discussed the limitations of studies relying on retrospective verbal reports (Leclair-Visonneau et al., 2010). A further limitation of verbal reports – which may explain inconsistent results - follows from the fact that visual targets are mostly selected automatically (i.e., without conscious attention) during visual scanning in wakefulness and probably in dreaming. This means subjects may only report consciously selected, qualitative content (Hong et al., 1997, 2009). However, studying persons with REM sleep behavior disorders may circumvent the limitations of studies relying purely on dream reports. Persons with this disorder enact dreams due to a failure to suppress muscle tone during REM sleep. Thus, directional correspondence of REMs with head and limbs during goal-oriented behavior can be observed directly. Indeed, studies of this sort suggest that “when present, REMs imitate the scanning of the dream scene” (Leclair-Visonneau et al., 2010).

Evidence against the scanning hypothesis (Arnulf, 2011) can be summarized as follows: (1) REMs may be unaccompanied by visual imagery in some cases of dreaming (e.g., in fetuses, neonates, congenitally blind, cats without visual cortex) and vice versa; visual imagery in dreaming may not always be accompanied by REMs (e.g., non-REM sleep, tonic REM sleep). The congenitally blind can report landscapes, objects and human figures in their dreams and are able to draw them. Furthermore, those dream content reports correlate with the decrease in alpha strength recorded from occipital regions (Bértolo et al., 2003). This suggests visual imagery in dreaming of the congenitally blind, because alpha activity attenuation is regarded as an indicator of visual imagery in general (Lopes da Silva, 2003). Furthermore, REM-locked activation in visual brain areas *in utero* suggests a REM-locked primitive form of vision (Schöpf et al., 2014). (2) In monkeys many binocular REMs are not conjugated (Zhou and King, 1997). This finding appears to be a potential argument against the scanning hypothesis, but it may indicate that binocular coordination is often disrupted during sleep – in much the same way as it is disrupted in wakefulness in cases of strabismus.

### Dreaming in Sleep Without REMs

Rapid eye movement sleep has often been equated with dreaming sleep, but visual dreams occur when REMs are not observed; i.e., in tonic REM sleep (Foulkes and Pope, 1973; Hodoba et al., 2008) and in non-REM sleep (Fosse et al., 2001; Siclari et al., 2013).

Our basic premise is that REMs offer a unique probe into consciousness (as opposed to representing an epiphenomenon). This argument rests upon the notion of active vision that has become essentially internalized during sleep (Hobson and Friston, 2014). The neurophysiological and anatomical evidence upon which this argument rests has been reviewed in detail in (Hobson and Friston, 2012). This evidence rests largely on the remarkable homology between PGO waves in REM sleep (Nelson et al., 1983) and the neurophysiological responses that accompany oculomotor reflexes during saccadic searches of a visual scene in active (visual) inference (Purpura et al., 2003). Clearly, associating REMs with qualitative experience during perceptual synthesis in REM sleep does not necessarily imply a one-to-one relationship between REM and dreaming. For example, neuromodulatory changes (Hobson, 2009) that disengage the oculomotor (pontine) correlates of internalized visual searches could, in principle, permit deep hierarchical inference in the absence of PGO waves and overt REMs. We mean this in the same sense that attention can, on some views, be seen as covert eye movements that are not accompanied by activation of the superior colliculus and pontine nuclei (Rizzolatti et al., 1987; Bernier et al., 2017). In other words, there is a possibility that qualitative visual experience could arise in non-REM sleep; in the same sense that we can deploy visual attention without actually moving our eyes. This speaks to the debate about the prevalence of dreaming during REM and non-REM sleep – see Arnulf (2011) for discussion on this central issue.

The potential dissociation between REM and dreaming also speaks to an interesting issue (raised by our reviewers) about scene construction from the first and second person perspectives (Hassabis and Maguire, 2007; Mirza et al., 2016). The crucial role of projective geometry in generative models of perspective taking has recently been acknowledged – and linked explicitly to active inference and predictive coding (Rudrauf et al., 2017). Second person perspectives are quintessentially counterfactual and would therefore not necessarily entail oculomotor responses. This counterfactual aspect is an inherent part of active inference during scene construction; namely, inferring “what would I see if I looked over there” (Mirza et al., 2016). This computational aspect of (counterfactual) predictive coding further suggests that REM indexes a particular aspect of dream phenomenology; namely, rehearsing an active visual engagement with the world – from a first-person perspective. It is this particular aspect of active perceptual synthesis that we propose underwrites the unique relationship between a particular sort of qualitative perceptual (conscious) experience and REMs.

The issues and controversies surrounding the neural correlates of non-REM dreaming have been discussed previously (Nielsen, 2000). These correlates are notoriously difficult to study because non-REM sleep lacks an easily detectable event for correlation (c.f., REMs in REM sleep). However, one would predict that non-REM sleep dreaming shares neural machinery with REM dreaming, for example, Nielsen (2000) proposed that “covert” REM sleep processing — during non-REM sleep — generates non-REM sleep mentation. Alternatively, Siclari et al. (2017) have reported that dream experience correlates with high density EEG measures of ‘activation’ in posterior cortical regions, whether the subjects are awakened from REM sleep or from non-REM sleep, whether they could recall the content of dream experience or not. Furthermore, seeing faces, places or movement in REM sleep dreams was associated with increased high-frequency EEG activity in specific cortical areas, which corresponded to those engaged with visual processing of the same contents in wakefulness (Siclari et al., 2017). Likewise, a machine-learning decoder — trained on human fMRI responses to natural images (e.g., male, car, and street) in wakefulness — suggests that the same neuronal representations are engaged to experience the same images in dreaming; during sleep-onset non-REM sleep (Horikawa et al., 2013).

In summary, EEG studies of the neural correlates of dreaming (Siclari et al., 2017) and fMRI studies of the neural correlates of REMs (Hong et al., 2009) speak to a common neural architecture for visual experience across REM sleep, non-REM sleep and wakefulness, but there are many unknowns in this area that need to be explored.

### Neural Correlates of REMs (Studied With fMRI) vs. Dreaming (Studied With EEG)

It seems safe to say that REMs are coupled with dreaming consciousness and particularly visual perception in dreaming, because fMRI evidence for REM-locked visual information processing and multisensory and sensorimotor integration is robust. This sort of integration is essential for waking consciousness and probably for dreaming consciousness as well (Metzinger, 2003, pp. 254, 258), and there are correlations between REMs and visual experience in dreaming that can be seen at the within-subject level (Hong et al., 1997). However, the neural correlates of REMs studied with fMRI (Hong et al., 2009) do differ from the neural correlates of dreaming studied with high-density EEG (Siclari et al., 2017). These differences may be explained by the measurements of brain activity: EEG has greater temporal resolution, while fMRI has superior spatial resolution; EEG electrodes are more sensitive to neuronal signals from the surface of the brain, in relation to deeper subcortical brain structures. There are also differences in independent variables; i.e., motor behavior versus the subjective dream experience: unlike dream experiences, REMs are temporally precise with a brief duration and well-defined onset and termination, thus enabling study of time-locked changes. Unlike REMs, dream experiences are more ambiguous and have varied content: Should a brief thought-like mentation be called a dream? Is its neural correlate the same as the neural correlate of a dream experience with a narrative? In principle, the time series of REMs should afford greater statistical power in the search for the neural correlates of dreaming, in relation to simply comparing neuronal responses in the presence or absence of dream experience.

An interesting issue in this setting is that not all awakenings from phasic REM sleep with REM bursts produce a dream report (Siclari et al., 2013). One explanation may be that conscious experience is a graded phenomenon (Metzinger, 2003, pp. 135, 604, 620); rather than simply absent vs. present – and not all dream experiences that are time-locked to REMs become conscious. Alternatively, dream experience simply could not be remembered, thus not reported (suggested by our reviewer).

To fully address these issues further studies are needed; including high-density EEG studies of neural correlates of REMs (possibly with reanalysis of collected data) and fMRI/MEG studies of the neural correlates of dreaming.

## REMs — Natural Task-Free Probe Ideal for Neurodevelopmental Studies

Visual perception — which may appear to be a passive process — is, on the active inference view, an active process of inferring the world that involves moving the eyes rapidly (about four times a second) to salient targets. We proposed that REMs index the task of generating and scanning the model of the world through multisensory integration in dreaming (Hobson et al., 2014). In other word, REMs co-occur with these important brain events which are essential for perception, both in dreaming and in wakefulness. Event-related fMRI (the events being video-timed REMs) takes snapshots of these fundamental mind/brain events.

Since REMs are natural, task-free probes, the study of REMs with fMRI can be used in many different subjects, including infants, persons with dementia, and animals, who cannot tolerate conventional brain activation paradigms (Hong and Harris, 2009; Hong et al., 2009). Furthermore, it is feasible to time REMs of the human fetus by calculating lens center and eye center on serial fMRI images. This means one can study REM-locked fetal brain activity using fMRI (Schöpf et al., 2014). Available fetal brain imaging (Ferrazzi et al., 2014; Dunn et al., 2015; Jakab et al., 2015; van den Heuvel and Thomason, 2016) also includes MEG. In short, longitudinal studies from before birth are, in principle, now possible.

Rapid eye movements occur in mammals and birds (Hobson, 2009) and Australian dragons (Shein-Idelson et al., 2016), thus REM-locked changes can be studied in other species than humans. For example, the sleeping dragon study showed robust correlation of these video-timed REMs with intracranial electrophysiological (including single cell) recordings (Shein-Idelson et al., 2016). Additionally, this study also showed that video recording of closed eyes can be rendered to computerized analysis to time and quantify REMs automatically.

In summary, REMs offer a promising probe to study phylogenetic and ontogenetic development of the basic mind/brain function, especially visual perception.

### Why Are Infants Ideal for Studying REMs?

Crucially, infants are ideal subjects for REM sleep studies because they sleep for several hours during the daytime; REM sleep is usually their initial sleep state; and up to half of their sleep (more than half in premature infants) is REM sleep (Hong and Harris, 2009). Furthermore, using REMs as a task-free probe that is not confounded by differences in task difficulty and subject performance — issues that often confound developmental studies — enables the matched comparison of findings from infants with adults throughout the entire life span. It is straightforward to study REMs in sleep in infants. Compared to studying REMs in adults, it is easier and less expensive to study REMs in neonates.

On a practical note, our previous neonate studies have demonstrated feasibility and safety. Notably, 2 h during daytime sufficed for collection of data from a neonate, whereas we reserved 16 h over two nights for an adult. One of the two neonates studied had sufficient REM sleep for analysis (the video recording of REMs of this neonate can be found in Supplementary Video S1). Event-related analysis revealed fMRI signal increases in the thalamus and signal decreases in the visual cortex time-locked to REMs. These findings are consistent with previous fMRI studies on sleeping (Born et al., 1998; Redcay et al., 2007) or sedated (Yamada et al., 1997, 2000) children, which show that photic stimulation through children’s closed eyelids induced a signal decrease in the visual cortex. Because REM sleep latency is 3–4 h for the age group 4 1/2 – 7 (Roffwarg et al., 1966), so two consecutive nights study may be required for this age group, as for adults.

### Why Should REMs Be Timed From Video-Recording Instead of EOG?

Electroculogram is the most commonly used method for monitoring eye movements. However, because rapidly changing magnetic fields during MRI confound the EOG signal, video-recording detects approximately four times as many REMs in fMRI studies compared to EOG recording (Hong et al., 2009). EOG measures electric currents induced by movements of the electrically charged eyeball, whereas video-monitoring directly measures eye movements (see Supplementary Video S2 for a video-recording of REMs during fMRI).

### Video-Timing Findings Lend Support to Predictive Coding

Crucially, fMRI correlates of REMs timed with EOG (Wehrle et al., 2005; Miyauchi et al., 2009) are similar to those with video-timing. However, it is our new findings in the video-timed study (Hong et al., 2009) that are construed as empirical support for predictive coding (Hobson et al., 2014). First, fMRI findings of video-timed REMs (Hong et al., 2009) endorse the premise that REMs generate and scan dream visual imagery, as mandated by ‘active inference’ under predictive coding (Hobson et al., 2014).

Second, video-timing REMs identify REM-locked activation of the subpallidal basal forebrain (Figure 5D), the major source of cholinergic input to the cortex (Hong et al., 2009). From the perspective of predictive coding, cholinergic modulation may play an important role in modulating and gating prediction error signals (Hobson et al., 2014). REM-locked cholinergic boosting of superficial pyramidal cells in the cortex is potentially important from the perspective of predictive coding, as superficial pyramidal cells (layers II and III) are thought to encode prediction error (Hobson et al., 2014).

Third, video-timed REMs showed that REM-locked activation occurs in the claustrum and thalamic reticular nucleus (Figure 5C) and the precentral motor and postcentral somatosensory cortex (Figure 5A) and the right RSC (Figure 4) (Hong et al., 2009). These fMRI findings are consistent with the idea that perception and action are intimately related (Friston, 2010; Friston et al., 2012; Brown et al., 2013; Clark, 2013). Converging evidence suggests that RSC may play a role in scene construction and spatial navigation (Hassabis and Maguire, 2007). Spatial navigation requires sensory-motor integration. REM-locked activation in the sensorimotor cortex is in line with intracranial EEG findings in human subjects (De Carli et al., 2016) and with fMRI findings in lucid dreamers (Dresler et al., 2011). REM-locked activation in the precentral motor - and postcentral sensory cortex - speaks to an intimate perception-action coupling in general as well as visual perception-oculomotor integration in particular. The thalamic reticular nucleus and claustrum are crucial for sensory-motor binding (Crick, 1984; Crick and Koch, 2005). In summary, fMRI correlates of video-timed REMs are consistent with the proposal that the brain is organized so as to facilitate sensory-motor binding (Engel and Singer, 2001) and scene construction during eye movements in both sleep and wakefulness. Thus, fMRI studies of video-timed REMs (as opposed to fMRI study of EOG-timed REMs) may offer a powerful tool to examine the functional brain architectures that underwrite active inference in wakefulness – and sleep.

## Active Inference and Protoconsciousness Hypothesis

Rapid eye movement sleep is most abundant during fetal development and infancy, then decreases gradually, but persists throughout life (Figure 1). This time course suggests that it serves an indispensable role, not only in the development of perceptual capacity (Marks et al., 1995; Schöpf et al., 2014), but also in maintaining it (Hobson, 2009). To articulate this from the perspective of predictive coding, “dreaming plays an essential role in maintaining and enhancing the capacity to model the world by minimizing model complexity and thereby maximizing both statistical and thermodynamic efficiency (Hobson et al., 2014)”.

In brief, perception entails perceptual inference and hypothesis testing. This means that it is necessary to distil general trends when fitting a model to noisy data – like the sensory data sampled from the world (Hobson et al., 2014). The resulting parsimony principle – of choosing the simplest explanation that provides an accurate account of the data at hand – is necessary; not only for scientific hypotheses but also perceptual hypotheses (Hobson and Friston, 2012; Hobson et al., 2014). Attending to every detail leads to a failure to generalize and make useful predictions about new data. In statistical inference and machine learning this problem is known as ‘overfitting’ (Hobson and Friston, 2012), which may provide a metaphor for the psychopathology of autism (Pellicano and Burr, 2012; Lawson et al., 2014; Van de Cruys et al., 2014) that we will return to later.

In this setting, REM sleep may be necessary to minimize the complexity of the brain’s ‘generative models’ (Hobson et al., 2014). The brain is sequestered from the world in REM sleep because of sensory input gating (Hobson et al., 2000). In REM sleep there are almost no externally derived sensory data for which an accurate explanation is required (Hobson et al., 2014). Thus, in REM sleep the brain “can ignore the accuracy part of model evidence” and “can focus on minimizing (model) complexity, using exactly the same hierarchical prediction error minimization that it uses during wakefulness” (Hobson et al., 2014). This provides a formal and unified account of dreaming and waking perception, using exactly the same neuronal processes.

Inferring the causes of sensory signals requires complex brain structures that embody a (generative) model of how sensations are generated. Computing an explanation for the sensorium entails an enormous amount of energetic work, that is, substantial energy consumption by the brain. Happily, minimizing model complexity (statistical efficiency) guarantees efficient energy consumption (Hobson et al., 2014). Although the brain strives for statistical and thermodynamic efficiency, it still consumes a substantial amount of energy: representing only about 2% of our body weight, our brain consumes about 20% of the body’s energy supplies. Mammals and birds that evince REM sleep can infer social stimuli which are “inherently more complex and ambiguous than non-social stimuli” (Pellicano and Burr, 2012). The imperative to reduce complexity during sleep may therefore be greater for the complicated brains of mammals and birds (Hobson and Friston, 2012; Hobson et al., 2014).

Protoconsciousness theory posits that sensorimotor integration is developed off-line *in utero* for a better chance of survival in outside world; for example, we practice walking during REM sleep *in utero*, before we actually learn to walk after birth (Hobson, 2017). This hypothesis is clearly difficult to prove, but is in line with the enhancement of procedural learning and memory associated with sleep in adults (Stickgold, 2005; Hobson, 2017). Procedural memory is used in automatic execution of the integrated procedures, like walking, swimming, driving a car or playing basketball, which are acquired only after repetitive training. Notably, sensorimotor integration time-locked to REMs in sleep was the key finding of our fMRI studies (Hong et al., 2009). Furthermore, robust REM-locked activation of RSC (Figure 4), the key structure engaged by spatial navigation (Maguire, 2001; Harker and Whishaw, 2004; Vann et al., 2009), supports Hobson’s proposal of off-line practice of walking (navigation) during REM sleep *in utero*. Additionally, it is in line with the hypothesis that when developing infants attain locomotive capacity REM sleep diminishes (Roffwarg et al., 1966). In summary, there is much evidence to suggest that virtual reality models and generative models in predictive coding are formally equivalent (Hobson and Friston, 2012, Friston, 2014; Hobson et al., 2014).

## Predictive Coding Accounts of Severe Mental Disorders

Predictive coding theory explains what might happen if inference goes awry. Predictive coding offers a coherent explanation for waking perception as perceptual inference — and explains the experiences of persons with autism (Pellicano and Burr, 2012; Friston et al., 2013, 2014; Gómez et al., 2014; Lawson et al., 2014; Quattrocki and Friston, 2014; Skewes et al., 2014; Van de Cruys et al., 2014) and schizophrenia in terms of false inference (Dima et al., 2009; Fletcher and Frith, 2009; Brown et al., 2013; Friston et al., 2014). In what follows, we briefly consider predictive coding in schizophrenia (autism) and how it relates to REMs.

### Schizophrenia

In predictive coding, ‘precision’ controls the relative influence of bottom-up prediction errors and top-down predictions at different levels in hierarchical schemes. Statistically, ‘precision’ means the inverse of the variance or degree of uncertainty. This means that prediction errors that are afforded more precision have a greater impact on belief updating (Friston, 2010). ‘Precision’ is thought to be encoded by the postsynaptic gain of superficial (layers II–III) pyramidal cells encoding ‘prediction error’ (Adams et al., 2013; Friston et al., 2014). “This gain corresponds exactly to the synaptic efficacy that underlies the adaptive changes in connection strengths” (Friston et al., 2013).

Aberrant neuromodulatory ‘precision’ control in predictive coding is consistent with nearly every synaptic or physiological theory of schizophrenia, including a dysfunction of dopaminergic and NMDA receptors, GABAergic abnormalities, and dysfunctional excitation-inhibition balance (Friston et al., 2014). Subtle changes in the delicate balance of ‘precision’ ascribed to top-down prior beliefs and bottom-up sensory evidence can have a profound effect on inference, resulting in false beliefs (Adams et al., 2013; Friston et al., 2014), which may account both for hallucinations and delusions in schizophrenia (Fletcher and Frith, 2009). “Believing and perceiving, although conceptually distinct, emerge as deeply mechanically intertwined” (Clark, 2013).

Core defects in predictive coding have been used to explain or simulate not only familiar psychotic symptoms like hallucinations, catatonia, delusions, and misattribution of agency (Fletcher and Frith, 2009; Adams et al., 2013; Friston et al., 2014), but also an array of laboratory findings in schizophrenia; such as abnormal smooth pursuit eye movements, reduced responses to oddball stimuli interspersed with repetitive stimuli (i.e., a failure to adequately predict sensory input renders everything surprising), a deficit in sensory attenuation during self-generated activity (Adams et al., 2013; Friston et al., 2014) and resistance to illusion (illusions occur when prior predictions override veridical sensory evidence, as in the hollow-mask illusion). Dima et al. (2009) suggested a weakening of top-down prediction and strengthening of bottom-up sensory evidence in schizophrenia during presentation of hollow faces.

Efference copy (or corollary discharge) is an internal copy (or transformation) of a motor signal that predicts the sensory consequences of movement and “tag[s] these stimuli as self-generated (Fletcher and Frith, 2009)” (see Box 1). Failure to attenuate the sensory consequences of action or speech in patients with schizophrenia may lead to “misattribution of self-generated actions [and speech] to others,” and delusions of being controlled and auditory hallucinations, respectively (Fletcher and Frith, 2009). A recent fMRI study suggested reduced sensory attenuation in individuals with schizophrenia, especially those with high hallucination scores (Shergill et al., 2014). Failed sensory attenuation also explains why individuals with schizophrenia with auditory hallucinations and passivity experience are more likely to be able to tickle themselves (Blakemore et al., 2000). Interestingly, it was reported that healthy individuals are more likely to self-tickle after awakening from a REM sleep dream (Blagrove et al., 2006).

### Autism

Predictive coding theory also provides an explanation for the broad range of phenomena in autism (Pellicano and Burr, 2012; Adams et al., 2013; Friston et al., 2014; Gómez et al., 2014; Lawson et al., 2014; Skewes et al., 2014; Van de Cruys et al., 2014), in particular in terms of an imbalance of precision toward sensory evidence (confidence placed in sensory evidence), relative to prior beliefs (Friston et al., 2014; Lawson et al., 2014). Aberrant precision has been used to explain various aspects of autism (Friston et al., 2014; Lawson et al., 2014); namely, reduced capacity for generalization (analogous to ‘overfitting’ in inferential statistics or to reduced ‘signal-to-noise ratio’), “extreme sensitivity to fluorescent lighting or to sound of the school bell, exceptional performance on the embedded figures test and finding hidden figures, an excellent eye for detail” and reduced susceptibility to visual illusion (Pellicano and Burr, 2012). Furthermore, the intense desire for sameness and repetitive behavior in autism may be a means of reducing the uncertainty in the environment (Pellicano and Burr, 2012).

Active inference and predictive coding formulations have been extended from exteroceptive sensations – like vision and audition – to interoceptive sensations from within the body (Seth, 2013; Quattrocki and Friston, 2014). In infants with autism, aberrant interoceptive inference may cause “a failure to contextualize interoceptive cues, elicited by maternal interactions,” a failure to engage with prosocial cues, and defective inference about others (Friston et al., 2014). Predictive coding theory may therefore provide a parsimonious explanation for social deficits in autism (Friston et al., 2014) as well as non-social symptoms and perceptual atypical behaviors (Pellicano and Burr, 2012). Autistic social withdrawal may be a means to avoid irreducible uncertainty about the environment and other people’s actions. In addition, many studies indicate impairment in multisensory integration in autism (Foss-feig et al., 2010; Russo et al., 2010; Hill et al., 2012). Crucially, our fMRI study of video-timed REMs was able to provide extraordinarily robust indication of REM-locked multisensory integration (and probably REM-locked generation of the model of the world via predictive coding). We conclude that fMRI study of video-timed REMs is a promising tool to study autism throughout development over the entire life span, starting from birth.

### REMs in Schizophrenia and Autism

What would one predict in terms of REMs in schizophrenia and autism? This is an interesting question from several perspectives. There is a substantial body of evidence that suggests people with schizophrenia and autism have difficulties modulating the precision of sensory signals (Friston, 2017) and in particular, their attenuation during movement (Shergill et al., 2005; Oestreich et al., 2015). One obvious prediction is that REM should be more prevalent due to a failure of sensory attenuation (and possible compensatory changes higher in the oculomotor hierarchy). To a certain extent, this is consistent with more exuberant REMs and REM sleep in schizotypy (Kuula et al., 2018) and autism, where comorbidity is associated with increased REM sleep pressure (Baglioni et al., 2016). The relationship between REM and schizophrenia is particularly interesting given that abnormalities of eye movements (particularly, slow pursuit) are one of the most robust soft neurological signs associated with schizophrenia. In predictive coding or active inference formulations, these signs implicate abnormal neuromodulation or precision control in the visual (and oculomotor) hierarchy (Adams et al., 2012).

## Conclusion and Future Directions

To recap, we have established a formal link between dream imagery, REM and the hierarchical, computational anatomy of cortical and subcortical systems in the brain, with a special focus on the PGO system. Much of the phenomenology of interest rests upon the gating or precision control of ascending prediction errors and descending predictions during wakefulness and dreaming. It is this connection between the phenomenology of perceptual synthesis and the physiology of neuromodulation that, in principle, motivates a link between conscious processing and the coordination of message passing in terms of neuromodulatory and attentional selection. This link speaks to a fundamental role for attention and mental action in perceptual inference; both in dreaming and wakefulness.

Our main assertion is that REMs provide a well-defined empirical entree into the neurophysiological mediation of qualitative experience. In this sense, there is a potential to use REM studies to ask whether disorders of consciousness and perceptual inference can be understood within this extended framework. We considered some important new leads that suggest aberrant precision in predictive coding can indeed be used to understand some key psychiatric disorders. REM-locked multisensory integration — as demonstrated by our fMRI studies — indicates REM-locked updating of the brain’s model of the world (and dreamer’s body at its center) through multisensory integration. The implicit multisensory binding is an integral part of multi-level hierarchical predictive coding. REMs in dreaming sleep can therefore be employed as a marker of this important mind/brain event. Studying the neural correlates of REMs may lead to a better understanding of the mind/brain and its aberrant function in people with autism and schizophrenia.

Magnetoencephalography (MEG) is a promising tool to study regional activity changes time-locked to REMs in sleep (Gott et al., 2017), in particular, multisensory-motor integration as well as PGO waves (Ioannides et al., 2004). MEG has good spatial resolution (millimeters precision) and a very high temporal resolution (milliseconds precision). MEG could show activity changes before and after REM onset in structures small in size (Ioannides et al., 2004). Furthermore, non-invasive brain imaging studies are well placed to identify brain structures for single unit studies. Finally, employing REMs as a probe may be particularly prescient in studies of neurodevelopment and psychopathology, enabling quantitative, non-invasive and neurophysiologically grounded studies of autism and schizophrenia - that are considered to be neurodevelopmental disorders. Longitudinal fMRI study of video-timed REMs from birth may shed light on fundamental questions about why our brain has developed consciousness and implicit self-modeling.

## Author Contributions

All authors listed have made a substantial, direct and intellectual contribution to the work, and approved it for publication.

## Conflict of Interest Statement

The authors declare that the research was conducted in the absence of any commercial or financial relationships that could be construed as a potential conflict of interest. The reviewer TA and handling Editor declared their shared affiliation.

## Supplementary Material

The Supplementary Material for this article can be found online at:

**VIDEO S1 |** Video-recording of rapid eye movements in sleep during our fMRI study (an adult) https://drive.google.com/file/d/0B0R_1dukpEw7QXpObTNZLWp0M3M/view?usp=sharing.

**VIDEO S2 |** Video recording of rapid eye movements in sleep during our fMRI study (a neonate) https://drive.google.com/file/d/0BzWLE2tIe0XhYXlBRnNRRHYtM2c/view?usp=sharing.

## Abbreviations

EOG: electroculogram
fMRI: functional magnetic resonance imaging
MTL: medial temporal lobe
PET: positron emission tomography
PGO: pontine-geniculate-occipital
REMs: rapid eye movements (not REM sleep)
RSC: retrosplenial cortex
