# Corrigendum: Rapid Eye Movements in Sleep Furnish a Unique Probe Into Consciousness

**DOI:** 10.3389/fpsyg.2018.02493

**Published:** 2018-12-10

**Authors:** Charles C.-H. Hong, James H. Fallon, Karl J. Friston, James C. Harris

**Affiliations:** ^1^Patuxent Institution, Correctional Mental Health Center — Jessup, Jessup, MD, United States; ^2^Department of Psychiatry and Behavioral Sciences, The Johns Hopkins Hospital, Baltimore, MD, United States; ^3^Department of Anatomy and Neurobiology, University of California, Irvine, Irvine, CA, United States; ^4^Department of Psychiatry and Human Behavior, University of California, Irvine, Irvine, CA, United States; ^5^The Wellcome Trust Centre for Neuroimaging, Institute of Neurology, University College London, London, United Kingdom

**Keywords:** predictive coding, dream, rapid eye movements (REMs) in sleep, autism, visual perception, retrosplenial cortex, claustrum, thalamic reticular nucleus

In the original article, there was an error. Crucially, fMRI correlates of REMs timed with EOG (Wehrle et al., 2005; Miyauchi et al., 2009) are similar to those with video-timing and have been construed as empirical support for predictive coding (Hobson et al., 2014).

A correction has been made to the first paragraph of the Sub-section Video-Timing Findings Lend Support to Predictive Coding.

Crucially, fMRI correlates of REMs timed with EOG (Wehrle et al., 2005; Miyauchi et al., 2009) are similar to those with video-timing. However, it is our new findings in the video-timed study (Hong et al., 2009) that are construed as empirical support for predictive coding (Hobson et al., 2014).

The authors apologize for this error and state that this does not change the scientific conclusions of the article in any way. The original article has been updated.

## Conflict of Interest Statement

The authors declare that the research was conducted in the absence of any commercial or financial relationships that could be construed as a potential conflict of interest.
